# Supplementary material for: Isolation and Characterization of the Arapaima gigas Growth Hormone (ag-GH) cDNA and Three-Dimensional Modeling of This Hormone in Comparison with the Human Hormone (hGH)
Source: Biomolecules. 2023 Jan 12;13(1):158. doi: 10.3390/biom13010158 (PMC9855374; doi:10.3390/biom13010158)

Figure S1. Comparison of Ramachandran plots and QMEAN scores of SWISS-MODEL's selected model, the two refined ones, and the model obtained from AlphaFold.

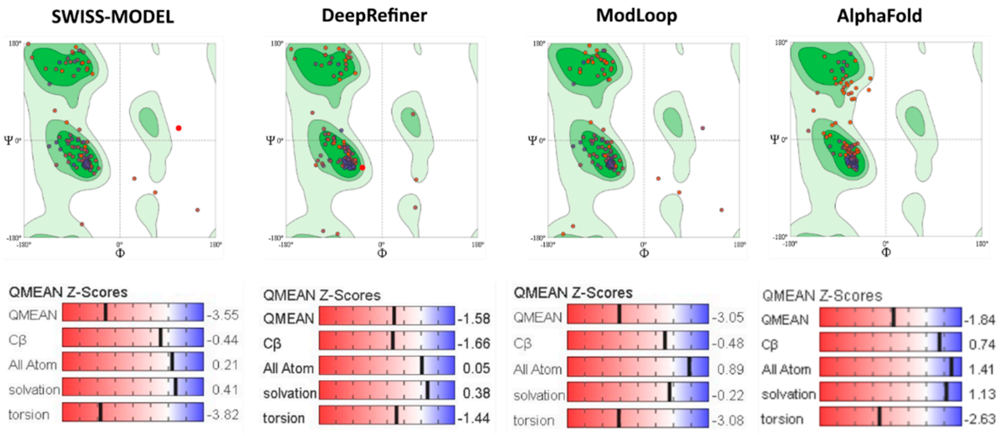

Supplement: Supplementary file 1 [file biomolecules-13-00158-s001.zip › biomolecules-2050753-supplementary.pdf]
